# Mitogen-activated protein kinase Hog1 is activated in response to curcumin exposure in the budding yeast Saccharomyces cerevisiae

**DOI:** 10.1186/s12866-014-0317-0

**Published:** 2014-12-19

**Authors:** Gajendra Kumar Azad, Vikash Singh, Mayur Jankiram Thakare, Shivani Baranwal, Raghuvir Singh Tomar

**Affiliations:** Laboratory of Chromatin Biology, Department of Biological Sciences, Indian Institute of Science Education and Research, Bhopal, 462023 India; Current address: Department of Genetics, Institute of Life Sciences, The Hebrew University of Jerusalem, Jerusalem, 91904 Israel

**Keywords:** Yeast drug response, Curcumin, HOG pathway, Hog1 phosphorylation, Glycerol-3-phosphate dehydrogenase 1 *(GPD1)*, Mitogen-activated protein kinases

## Abstract

**Background:**

Curcumin (CUR), an active polyphenol derived from the spice turmeric, has been traditionally used for centuries in ancient Indian medicine to treat a number of diseases. The physiological effects of CUR have been shown to be diverse; however, the target molecules and pathways that CUR affects have yet to be fully described.

**Results:**

Here, we demonstrate for the first time that the budding yeast mitogen-activated protein kinase (MAPK) Hog1 is essential for the response to CUR. Moreover, CUR-induced Hog1 phosphorylation was rescued by supplementation of iron to the growth medium. Hog1 was rapidly phosphorylated upon CUR treatment, but unlike the response to hyperosmotic shock (0.8 M NaCl), it remains activated for an extended period of time. A detailed analysis of HOG pathway mutants revealed that Pbs2p, Ptc2p, and Ssk2p are required for optimal CUR-induced Hog1 phosphorylation. We also observed a Hog1 dependent transcriptional response to CUR treatment that involved the up-regulation of glycerol-3-phosphate dehydrogenase 1 *(GPD1)*, a factor that is essential for the hyperosmotic stress response.

**Conclusions:**

Our present finding revealed the role of Hog1 MAPK in regulation of CUR-induced transcriptional response. We anticipate that our finding will enhance the understanding on the molecular mode of action of CUR on *S. cerevisiae*.

## Background

Yeast cells have evolved sophisticated mechanisms to withstand a variety of stresses including limited availability of nutrients, fluctuations in temperature, changes in osmolarity, and the presence of harmful agents such as radiation or toxic chemicals. A myriad of strategies have evolved to maintain cellular homeostasis under stressful conditions including the activation of mitogen-activated protein kinase (MAPK) pathways. Thus far, 5 MAPK pathways have been characterized in *S. cerevisiae* [1]*.* The fundamental function of these MAPK pathways is to regulate gene expression in response to various extracellular signals.

The high osmolarity glycerol (HOG) pathway is one of the most thoroughly studied MAPK pathways in yeast. The HOG pathway involves the MAPK Hog1 that specifically responds to increased extracellular osmolarity and is essential for cell survival under these conditions. Yeast cells respond to osmotic stress by activating Hog1 phosphorylation and, subsequently, translocating Hog1 to the nucleus where it directly interacts with several transcription factors to modulate gene expression. Recently, several studies have demonstrated additional functions of the HOG MAPK pathway. Evidence shows that the HOG pathway is essential for regulating the stress adaptation response induced by heat [2], citric acid [3], or low temperature [4]. The HOG pathway is also involved in providing tolerance to methylglyoxal [5] and the bacterial endotoxin lipopolysaccharide (LPS) [6], and reportedly plays a role in cell wall maintenance [7] and the distribution of proteins within the Golgi [8].

Curcumin (diferuloylmethane) is the principal bioactive agent in the spice turmeric [9]. Turmeric contains a class of compounds known as the curcuminoids, which includes curcumin, desmethoxycurcumin and bisdesmethoxycurcumin [10]. CUR has been consumed as a dietary supplement for centuries and has been widely used in ayurvedic medicines [11]. Because of the promising therapeutic potential of CUR, several clinical trials have been initiated or conducted to explore the effect of dietary CUR in the prevention of neurodegenerative diseases, several forms of cancer including colon and pancreatic cancer, bowel diseases, and other diseases [12-15]. Although extensive research has been performed on this drug, new biological targets of CUR are still being identified. In previous work, we demonstrated that the medicinal properties of CUR are largely the result of its cumulative effect on iron starvation and epigenetic modifications [16]. Thus, the present study was designed to test whether the Hog1 MAPK is also activated in yeast cells exposed to the natural compound CUR or not.

We demonstrate that CUR exposure in yeast cells leads to phosphorylation of Hog1 and up-regulation of GPD1 mRNA levels. The findings presented here strongly indicate that the ability of CUR to induce the osmoresponse may underlie many of the therapeutic activities of CUR.

## Methods

### Reagents and yeast strains

Unless otherwise stated, all the chemicals were purchased from Sigma-Aldrich. Curcumin (C1386), Bathopenanthrolinebisulfonic acid- BPS (B1375) and, FeSO_4_.7H_2_O (Sigma; F8263) were purchased from Sigma-Aldrich. To make synthetic complete (SC) media, amino acids, yeast nitrogen base (YNB), and ammonium sulfate were mixed together as per standard protocol [17]. Yeast cells were grown at 28°C in SC media supplemented with 2% dextrose (SCD). The *Saccharomyces cerevisiae* strains used in this study are listed in Table 1.

**Table 1:** **List of yeast strains used in this study**

**S.No.**	**Strain name**	**Genotype**	**Mutation**	**Source/Lab**
1	WT-15884C	MATa ade2-1 can1-100 his3-11,15 leu2-3,112 trp1-1 ura3-1	WT	Toshi Tsukiyama
2	WT	w303 MATa	WT	Erin K O’Shea lab
3	Hog1-GFP	EYO 690, Hog1-GFP(His) Nhp6a-RFP (KanMX6) MATa	HOG1-GFP	Erin K O’Shea lab
4	WT 4743	MATa/α his3Δ1/his3Δ1 leu2Δ0/leu2Δ0 LYS2/lys2Δ0 met15Δ0/MET15 ura3Δ0/ura3Δ0	WT	Yeast deletion collection-Open Biosystems (YDC-OB)
5	*hog1Δ/hog1Δ*	Isogenic to BY4743 *hog1Δ*::KANMX4	*hog1Δ*	YDC-OB
7	*pbs2Δ/pbs2Δ*	Isogenic to BY4743 *pbs2Δ*::KANMX4	*pbs2Δ*	YDC-OB
8	*ssk1Δ/ ssk1Δ*	Isogenic to BY4743 *ssk1Δ*::KANMX4	*ssk1Δ*	YDC-OB
9	*sho1Δ/sho1Δ*	Isogenic to BY4743 *sho1Δ* ::KANMX4	*sho1Δ*	YDC-OB
10	*msb2Δ/msb2Δ*	Isogenic to BY4743 *msb2Δ*::KANMX4	*msb2Δ*	YDC-OB
11	*ssk2Δ/ssk2Δ*	Isogenic to BY4743 *ssk2Δ*::KANMX4	*ssk2Δ*	YDC-OB
12	*ssk22Δ/ssk22Δ*	Isogenic to BY4743 *ssk22Δ* ::KANMX4	*ssk22Δ*	YDC-OB
13	*ste50Δ/ste50Δ*	Isogenic to BY4743 *ste50Δ*::KANMX4	*ste50Δ*	YDC-OB
14	*ptc1Δ/ptc1Δ*	Isogenic to BY4743 *ptc1Δ*::KANMX4	*ptc1Δ*	YDC-OB
15	*ptc2Δ/ptc2Δ*	Isogenic to BY4743 *ptc2Δ* ::KANMX4	*ptc2Δ*	YDC-OB
16	*ptc3Δ/ptc3Δ*	Isogenic to BY4743 *ptc3Δ*::KANMX4	*ptc3Δ*	YDC-OB

### FACS analysis of yeast cells

FACS analysis was performed as described earlier [18,19]. Briefly, yeast cells in the exponential growth phase were treated with alpha-factor to synchronize them in the G1 phase. Cells were released into media containing DMSO (control) or CUR (50 or 100 μM) at regular intervals for 6 h. Samples were collected and harvested by centrifugation. Ethanol was added to the cell pellets and they were vigorously vortexed. Samples were then centrifuged and washed once with 50 mM sodium citrate buffer (pH 7.0). RNase A was added to the samples and they were incubated at 37°C for 1 h. RNase A-treated samples containing 20 mg/ml propidium iodide (Sigma) were transferred to a BD FACS flow cytometer. DNA was detected using a BD FACS Aria III and analyzed using BD FACS Diva software.

### Nuclear-cytosolic extracts preparation

The spheroplasts were made from yeast cells as described earlier [20]. spheroplasts were washed once with ice-chilled wash buffer (100 mM KCl, 50 mM HEPES-KOH pH 7.5, 2.5 mM MgCl_2_, and 0.4 M Sorbitol) and lysed in lysis buffer (20mMHEPES at pH7.5, 50mMNaCl, 1mMEDTA, 0.1%Tween 20, 1mM phenylmethylsulphonyl fluoride, protease inhibitor cocktail) with 8 strokes in a chilled tight-fitting pestle dounce homogenizer, after 15min incubation on ice. Unbroken cells and debris were removed by centrifugation for 5min at 300g at 4°C. The cytoplasmic fraction (supernatant) was collected after a spin at 13000g for 20 min at 4°C and the nuclear-enriched fraction (pellet) was washed once with lysis buffer before collection. Normalized volumes of cytoplasmic fraction and nuclei fraction were then subjected to western-blotting analysis.

### Protein extraction and western blot analysis

Whole cell extracts from untreated and CUR-treated samples were prepared using the trichloroacetic acid (TCA) extraction method as described previously [21]. Western blotting was conducted following protocol used previously [22,23]. IRDye 700CW anti-rabbit IgG (diluted 1:15 000; LICOR Biosciences) was used as a secondary antibody. Blots were scanned using the Odyssey Infrared Imager (LI-COR Biosciences). The following primary antibodies were used: anti-GFP (Sigma, G1544), anti-p38/phospho-Hog1 (Cell Signaling, #92115), and polyclonal antibodies against recombinant yeast TATA binding protein (TBP) was raised in rabbit (Bhat Bio-tech India (P) Ltd.).

### Isolation of total RNA and real-time PCR

Exponentially growing wild type yeast cells or *hog1Δ* cells were treated with CUR (100 μM). Total RNA was isolated at the indicated time points (0, 30, 60, and 120 min) using the heat/freeze phenol method described elsewhere [24]. 1μg of total RNA was reverse transcribed to synthesize cDNA using the High Capacity RNA-to-cDNA Kit (Bio-Rad) according to the manufacturer’s instructions. Real time PCR experiments were performed using SYBR Green Mix (Roche diagnostics, USA) in an ABI real-time PCR machine. The primers used in this study are listed in Table 2.

**Table 2:** **List of primers used in the present study**

**S.no.**	**Gene**	**Forward primer sequence (5′-3′)**	**Reverse primer sequence (5′-3′)**
1	*HOG1*	GGATGCCTTGGCTCATCCTT	TGGTCATCAAACGTGGCAGA
2	*GPD1*	CATTGCCACCGAAGTCGCTC	AACCACAACCTAAGGCAACAACG
3	*ALG9*	TGCATTTGCTGTGATTGTCA	CAGGCAGTGGGAAATTCAGT

## Results

### CUR induced growth arrest in yeast cells was rescued by iron supplementation

**Figure 1:**
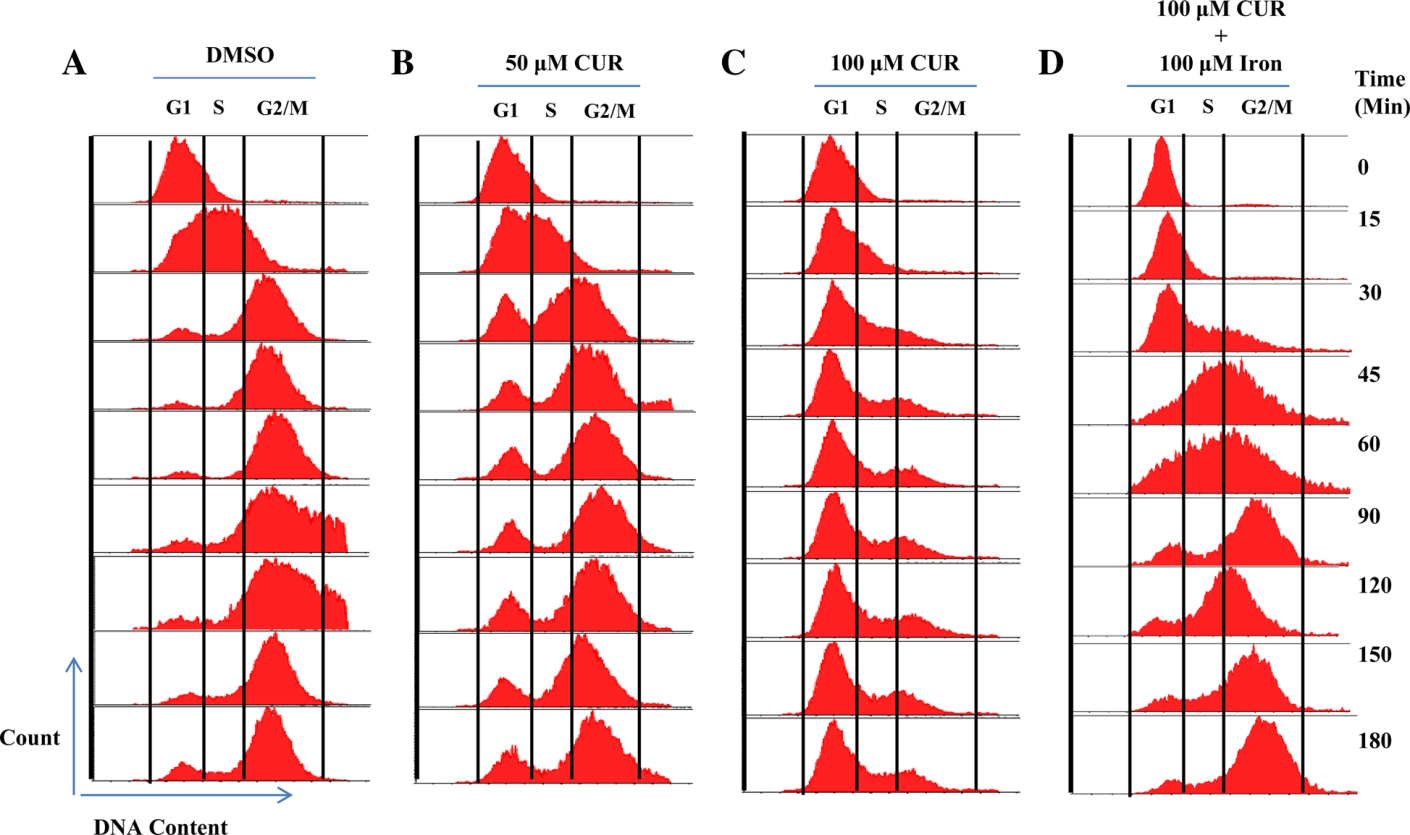
**CUR treatment induces growth arrest in yeast cells can be rescued upon iron supplementation.** Wild type cells (WT-1588-4C) were cultured in synthetic complete (SC) medium until they reached the exponential phase. Yeast cells were then treated with alpha-factor to synchronize them in the G1 phase. After synchronization, cells were released into media supplemented with **(A)** DMSO (control), **(B)** 50 μM CUR, **(C)** 100 μM CUR and **(D)** 100 μM CUR supplemented with 100 μM Iron. The cultures were sampled at the indicated time points and their DNA content was then analyzed by FACS.

To test the dose dependent effect of CUR on yeast cell cycle progression, we performed FACS analysis. Exponentially growing yeast cells were synchronized in the G1 phase using alpha-factor. After synchronization, G1 arrested cells were released into DMSO (control) or CUR (50 or 100 μM) supplemented media. The results from the FACS analysis revealed that the DMSO-treated cells quickly moved to the G2 phase within 30 minutes of release from the alpha-factor arrest (Figure 1A), whereas 50 μM CUR treatment led to a delay in the cell cycle progression (Figure 1B). In the case of 100 μM CUR treatment, cells remained in the G1 phase throughout the duration of the experiment (Figure 1C). Even after 360 minutes, cells released into media containing 100 μM CUR were not able to progress to the G2 phase (Figure 1C), suggesting that CUR causes prolonged G1 phase leading to delay in cell cycle progression. Previously, we have shown that iron supplementation rescues the growth inhibitory effect of CUR on yeast cells [25], hence we also analyzed the cell cycle progression in presence of curcumin after supplementation with iron. The results revealed that yeast cells recover from the cell cycle arrest in presence of iron (Figure 1D). However, the dose of iron used in this experiment (100μM) doesn’t affect cell cycle progression (data not shown). Altogether, these results indicate that CUR induced growth arrest in yeast cells can be rescued by iron supplementation.

### Hog1 is phosphorylated in response to curcumin treatment

**Figure 2:**
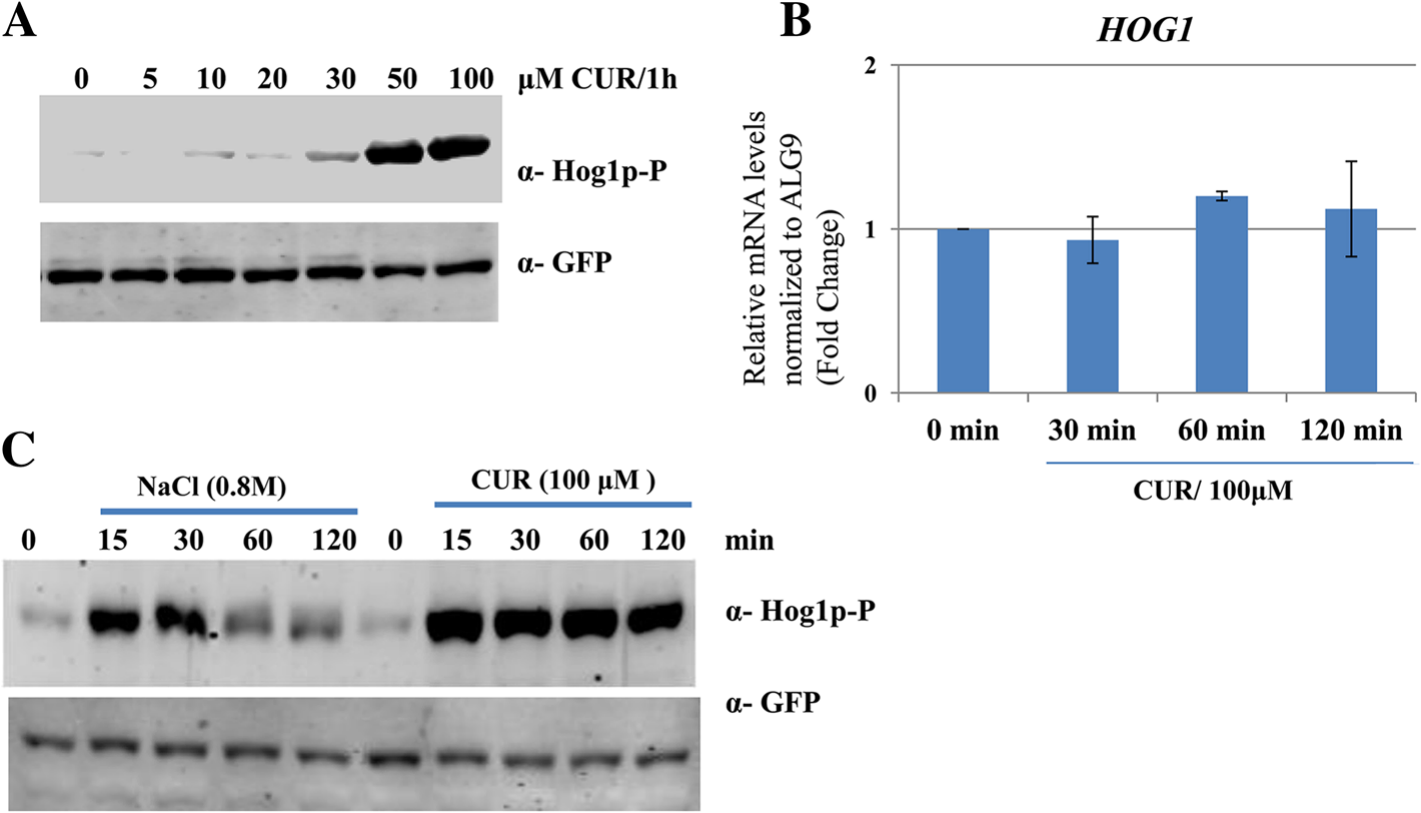
**Hog1 protein is phosphorylated in response to curcumin treatment. (A)** A yeast strain expressing GFP-tagged Hog1 (Hog1-GFP) was grown until the exponential phase. Protein was extracted from cells incubated for 1 h with increasing concentrations of CUR (0, 5, 10, 20, 50, and 100 μM). The phosphorylated form of Hog1 was detected using an anti-phospho-p38 antibody (phospho-Hog1). The western blot membranes were probed for total Hog1 using a polyclonal anti-GFP antibody and this served as a loading control. **(B)** An exponentially growing wild type yeast strain was exposed to 100 μM CUR for 2 h. Samples were taken after 0, 30, 60, and 120 min of incubation. The expression of *HOG1* mRNA levels was examined by SYBR Green real-time PCR. The error bars represent the standard deviation (SD) of three independent replicates. **(C)** Protein was extracted from cells incubated with either 0.8 M NaCl or 100 μM CUR at the indicated time points. The western blot membranes were probed for phospho-Hog1 or total Hog1 (anti-GFP). The anti-GFP signal served as a loading control.

Previously, we have identified several histone acetyltransferases (HATs) and histone deacetylases (HDACs) that are required to provide tolerance to CUR-induced stress [25]. One such HDAC, Rpd3p, is known to be involved in the transcriptional regulation of osmoresponse genes under conditions of osmotic stress [26]. Because *RPD3* deletion mutant was hypersensitive to CUR [25], we hypothesized that CUR might be inducing osmotic stress. To test this possibility, we analyzed the phosphorylation of Hog1, the central kinase involved in the regulation of osmoresponse in yeast. The phosphorylation of Hog1 is a hallmark for the activation of the osmotic stress response in yeast. To determine whether the exposure of yeast cells to CUR results in the activation of Hog1 protein, we analyzed the activation of Hog1 by performing a western blot with an antibody specific for the dually phosphorylated form of Hog1 (Thr-174/Tyr-176). Upon incubation with increasing concentrations of CUR (5–100 μM) for 1 h, we were able to detect the phosphorylated form of Hog1 at a dose of 50 or 100 μM CUR through western blot (Figure 2A). To determine that the observed increase in phosphorylated Hog1 levels is the result of phosphorylation and not the result of an increase in Hog1, we examined the total levels of Hog1. In these experiments, we used yeast cells that express GFP-tagged Hog1 (Hog1-GFP). As illustrated in the western blot using an anti-GFP antibody, we found no increase in the band density in response to CUR exposure (Figure 2A). To further substantiate these results, we measured *HOG1* mRNA levels upon exposure to CUR. As shown in Figure 2B, we failed to observe a significant increase in *HOG1* expression in response to CUR treatment.

Next, we were interested to analyze the time point kinetics of CUR-induced Hog1 phosphorylation. As a positive control, 0.8M NaCl treated samples were analyzed for Hog1 phosphorylation and as per reports [27-29] phosphorylation of Hog1 started very early and remained detectable for 30 min, after which it started to decrease (Figure 2C). While in case of CUR treatment, it was possible to detect phosphorylated Hog1 within 15 minutes of incubation. Interestingly, unlike NaCl, the CUR-induced phosphorylated Hog1 remained phosphorylated for an extended duration (at least 120 min) as revealed by western blotting (Figure 2C). Taken together, these data clearly suggest that CUR exposure induces Hog1 phosphorylation.

### Iron supplementation rescues curcumin-induced Hog1 phosphorylation

**Figure 3:**
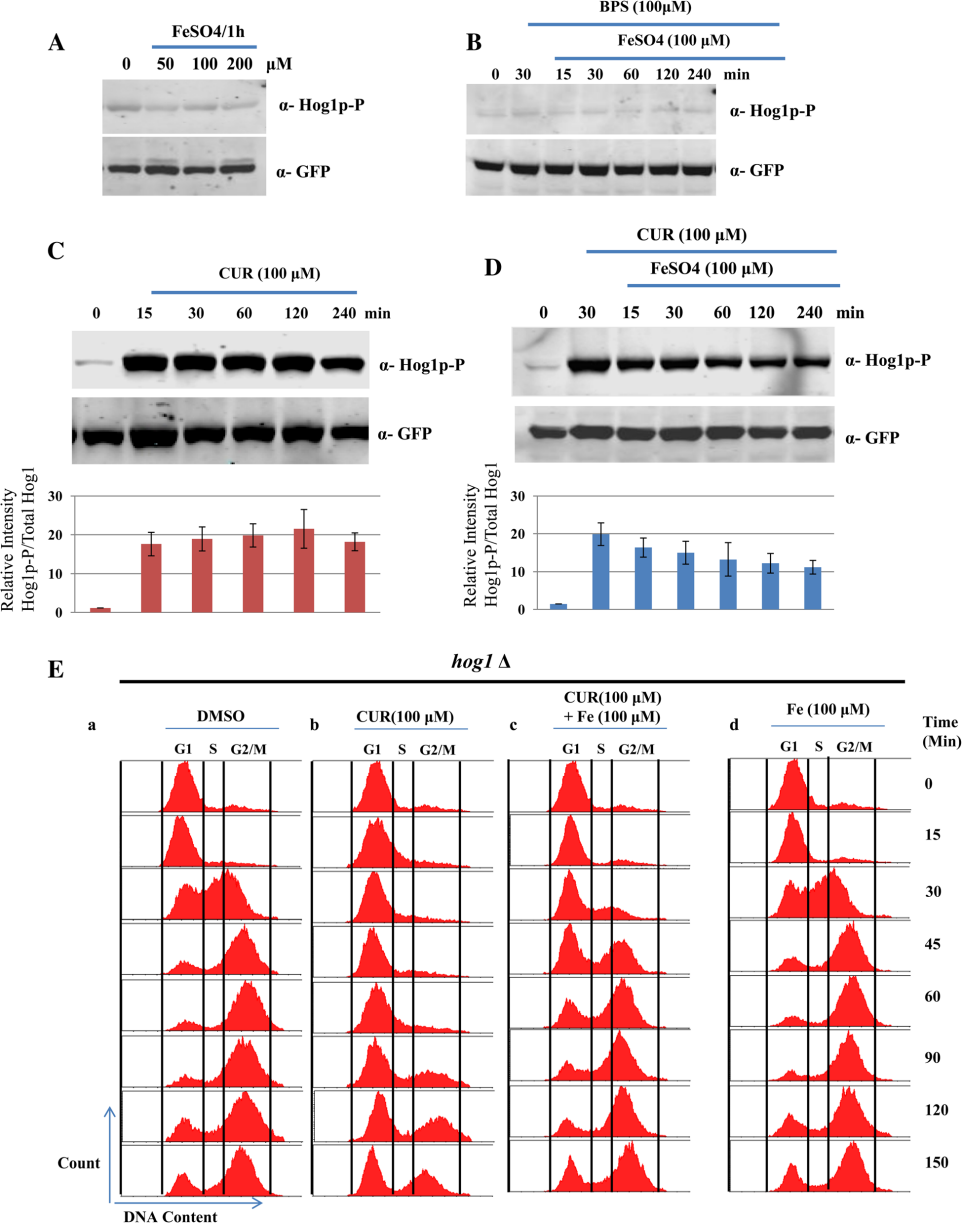
**Analysis of Hog1 phosphorylation after addition of iron. (A)** A yeast strain expressing GFP-tagged Hog1 (Hog1-GFP) was grown until the exponential phase. Protein was extracted from cells incubated for 1 h with increasing concentrations of iron (50, 100 and 200 μM/1h). The phosphorylated form of Hog1 was detected using an anti-phospho-p38 antibody (phospho-Hog1). The western blot membranes were probed for total Hog1 using a polyclonal anti-GFP antibody and this served as a loading control **(B)** Cells were treated with BPS for 30 min followed by addition of iron (100 μM) and cells were harvested at indicated time points. Proteins were extracted and phosphorylation of hog1 was detected by phospho-hog1 antibody. The western blot membranes were probed for total Hog1 using a polyclonal anti-GFP antibody and this served as a loading control **(C)** Protein was extracted from cells incubated with 100 μM CUR at the indicated time points. The western blot membranes were probed for phospho-Hog1 and anti-GFP (Total Hog1p). The intensity of phosphorylated Hog1 was quantified using Image J software and normalized with respect to total Hog1p levels (anti-GFP) and shown in the form of bar diagramme. The error bars represent the standard deviation (SD) of three independent replicates. **(D)** Yeast strain expressing GFP-tagged Hog1 (Hog1-GFP) was treated with 100 μM CUR for 30 min followed by addition of iron (100 μM). Cells were harvested at indicated time points in figure and protein were extracted. The phosphorylation of hog1 was detected by phospho-hog1 antibody. The western blot membranes were probed for total Hog1 using a polyclonal anti-GFP antibody and this served as a loading control. The intensity of phosphorylated Hog1 was quantified using Image J software and normalized with respect to total Hog1p levels (anti-GFP) and shown in the form of bar diagramme. The error bars represent the standard deviation (SD) of three independent replicates. **(E)** *Hog1*Δ cells were treated with alpha-factor to synchronize them in the G1 phase. After synchronization, cells were released into media supplemented with **(a)** DMSO (control), **(b)** 100 μM CUR, **(c)** 100 μM CUR supplemented with 100 μM Iron and **(D)** 100 μM Iron. The cultures were sampled at the indicated time points and their DNA content was then analyzed by FACS.

Since, we have observed that iron supplementation can rescue the growth inhibition caused by CUR treatment (Figure 1). Therefore, we examined whether the supplementation of iron can rescue the CUR-induced Hog1 phosphorylation. First, we analyzed the effect of Iron (FeSO_4_) and BPS (Iron chelator) on Hog1 phosphorylation. As shown in Figure 3A and B, iron or BPS doesn’t causes phosphorylation of Hog1, as no signal appeared in western blotting with phospho-Hog1 antibody. Next, we treated yeast cells with 100 μM CUR to observe the Hog1 phosphorylation till 4 h. We quantified the Hog1 phosphorylation levels using image J software and results revealed that phosphorylation remains almost constant till 4 h (Figure 3C, lower panel, bar diagram). Next, we treated exponentially growing yeast cells with 100 μM CUR for 30 min followed by supplementation of iron (100 μM) in the media. Cells were harvested till 4h post-treatment with iron. Our western blotting revealed that the levels of phosphorylated Hog1 was reduced significantly after addition of iron (Figure 3D, lower panel, bar diagram). These results clearly suggest that CUR-induced iron starvation leads to Hog1 phosphorylation and that can be rescued upon iron supplementation.

Since, we have observed the phosphorylation of Hog1 in response to CUR exposure; next we were motivated to observe the effect of CUR on HOG1 deletion mutant. We arrested *hog1*Δ cells with alpha-factor to arrest them in G1 phase as described in materials and methods. G1 arrested *hog1*Δ cells were released in fresh media containing DMSO or media supplemented with 100 μM CUR. The results from the FACS analysis revealed that the DMSO-treated cells quickly moved to the G2 phase within 30 minutes of release from the alpha-factor arrest (Figure 3Ea), whereas 100 μM CUR treatment led to a delay in cell cycle progression (Figure 3Eb). While, the cell cycle progression was resumed in presence of curcumin after supplementation with iron (Figure 3Ec). The results revealed that *hog1*Δ yeast mutant can also recover cell cycle arrest in presence of iron. The dose of iron used in this experiment (100μM) doesn’t affect cell cycle progression (Figure 3Ed). Altogether, these results indicate that CUR induced growth arrest in yeast cells can be rescued by iron supplementation.

### SSK2, MSB2, and PTC2 deletions abrogate curcumin-induced Hog1 phosphorylation

**Figure 4:**
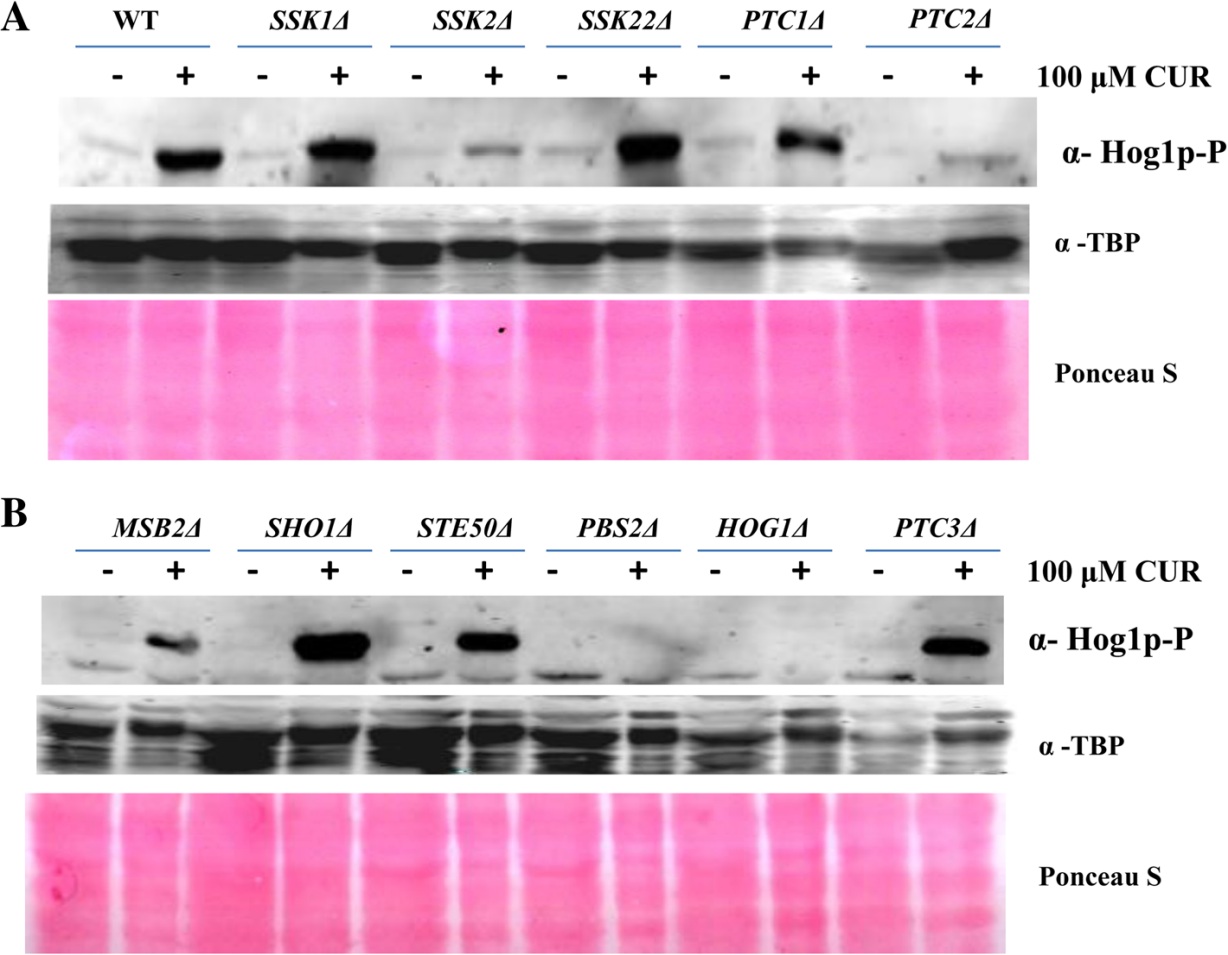
**Analysis of Hog1 phosphorylation in response to CUR treatment in HOG pathway mutants. (A and B)** Wild type and HOG pathway mutant yeast strains were grown in SC media until they reached the exponential phase. Cells were treated with 100 μM CUR for 1 h and protein was extracted as described in the Materials and Methods. The western blot membranes were probed for phospho-Hog1. As a loading control, the same blots were reprobed with anti-TBP antibody. Equal protein loading was further confirmed by Ponceau S staining of the western blot membrane.

As a member of MAPK family, Hog1 is phosphorylated by several upstream kinases that are involved in mediating osmostress response. We evaluated the phosphorylation of Hog1 protein in several HOG pathway mutants to investigate the role of these factors in mediating CUR-induced Hog1 phosphorylation. Wild type and mutant yeast strains were grown until they reached the exponential phase before being treated with 100 μM CUR for 1 h. Whole cell extracts were prepared as detailed in the Materials and Methods and a western blot was performed to analyze the phosphorylation of Hog1. As a control, cells were treated with the same concentration of DMSO. The western blot revealed that the phosphorylation of Hog1 was reduced in the *ssk2Δ, msb2Δ, ptc2Δ* and *pbs2Δ* deletion mutants relative to that in wild type cells (Figure 4A and B). These results suggest that Ssk2p, msb2p, Ptc2p, and Pbs2p are important components of the HOG pathway and are required for the optimal activation of Hog1 in response to CUR-induced stress.

### CUR-induced phosphorylated Hog1 gets translocated to the nucleus leading to over-expression of GPD1 mRNA levels

**Figure 5:**
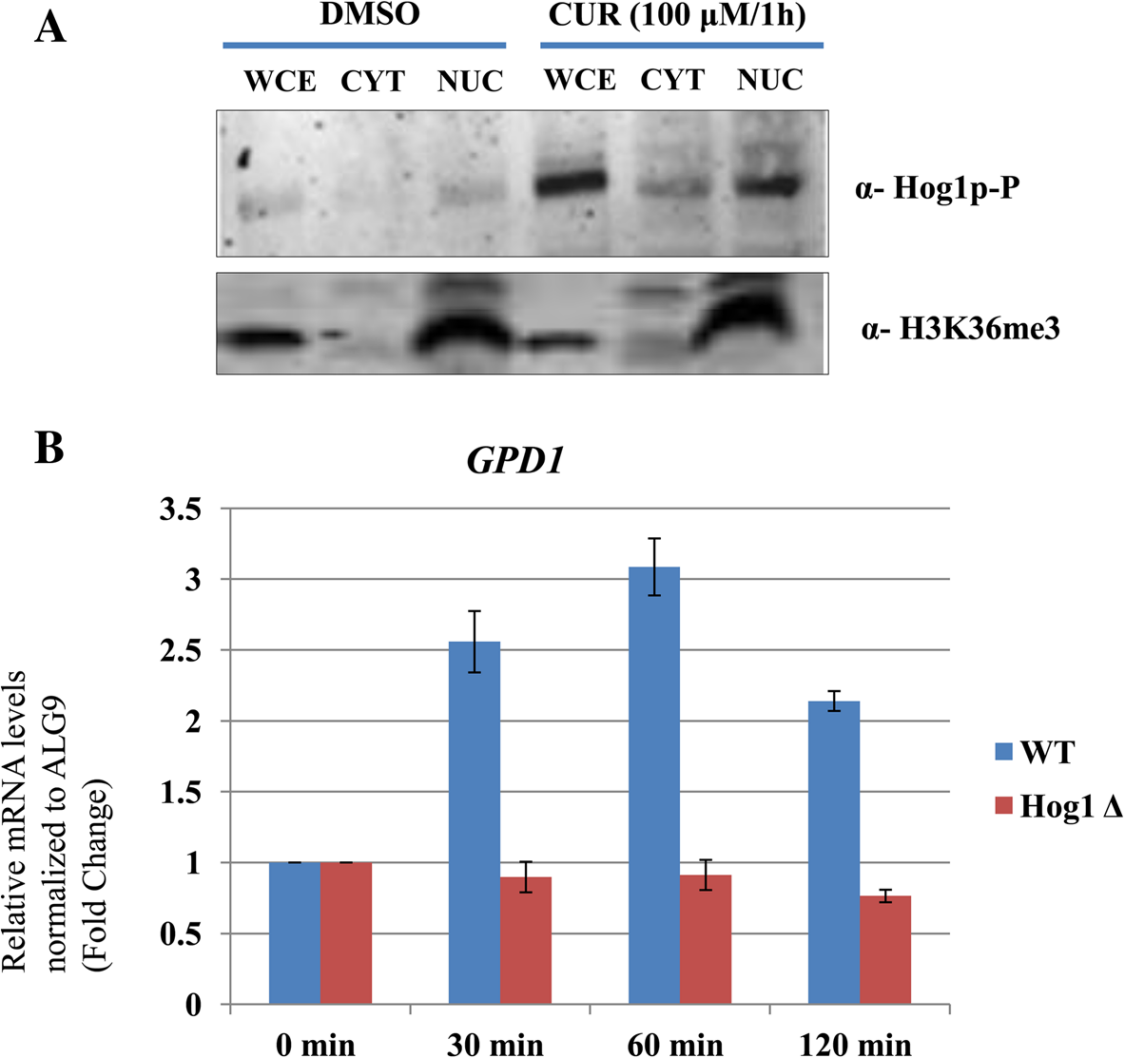
**CUR-induces Hog1 phosphorylation dependent over-expression GPD1 mRNA.** **(A)** Nuclear-cytoplasmic extracts were made as described in materials and methods. Whole cell extract (WCE), cytoplasmic fraction (CYT) and nuclear fraction (NUC) were loaded on SDS-PAGE and transferred onto nitrocellulose membrane. Western blot with anti-H3K36me3 antibody was performed to ensure the integrity of extract fractionation (H3K36me3 is a nuclear protein). The distribution of phosphorylated Hog1 was analyzed by probing with phospho-hog1 antibody. **(B)** An exponentially growing wild type or *hog1Δ* yeast strain was exposed to 100 μM CUR for 2 h. Samples were taken after 0, 30, 60, and 120 min of incubation. The expression of *GPD1* mRNA levels in wild type or *hog1Δ* cells was examined by SYBR Green real-time PCR. The fold-change in *GPD1* mRNA levels was normalized to the reference gene *ALG9*. The error bars represent the standard deviation (SD) of three independent replicates.

In response to osmotic stress, Hog1 is phosphorylated and translocated into nucleus where it regulates the expression of several osmoresponsive genes [30,31]. Hence, we were interested to determine the localization of CUR-induced phosphorylated Hog1. We treated exponentially growing yeast cells with 100 μM CUR for 1 h. We fractionated whole cell extract of yeast into nuclear and cytosolic fractions following a protocol as described in materials and methods. Our western blotting with phospho-hog1 antibody revealed that phosphorylated hog1 protein is localized into the nuclear fraction (Figure 5A). This result suggests that CUR-induced phosphorylated Hog1 is translocated to nucleus. It is well established that Hog1 is phosphorylated and translocated to nucleus in response to osmotic stress. Once it is in the nucleus, Hog1 regulates the expression of various osmoresponse genes [30,31]*.* This prompted us to analyze the expression of one such gene, *GPD1* that encodes a factor involved in glycerol biosynthesis during osmotic stress [32]. Wild type or *hog1Δ* yeast cells were treated with CUR (100 μM) and harvested at regular intervals (0, 30, 60, and 120 min) after treatment. Total RNA was extracted, reverse-transcribed to cDNA, and the expression of *GPD1* was analyzed using real-time PCR. Transcript levels were normalized to the reference gene *ALG9*. In case of wild type cells we observed a consistent 2–3 fold increase in the expression of *GPD1* within 30 min of CUR exposure (Figure 5B). This increase was maintained for 2 h (Figure 5B). This strongly indicates that upon CUR exposure, Hog1 is not only phosphorylated, but also causes an up-regulation of *GPD1* mRNA levels. To ensure that the up-regulation of GPD1 upon CUR treatment is due to Hog1, we analysed the expression of GPD1 mRNA in *hog1Δ* cells*.* Our quantitative PCR revealed that *hog1Δ* cells failed to up-regulate GPD1 mRNA upon CUR treatment (Figure 5B) suggesting the specific requirement of Hog1. Altogether, these results suggest that the CUR-induced transcription response requires Hog1 and mediated through Hog1 phosphorylation in yeast cells.

## Discussion

The diverse pharmacological activities of CUR have been attributed to its actions on multiple cellular targets, either by interacting physically with the targets themselves or by modulating transcription factors, enzyme activity, or gene expression. In this study, we examined the requirement for a functional HOG pathway to cope with CUR-induced stress. If signaling through Hog1 is essential for its function under CUR-induced stress, Hog1 should be activated by phosphorylation of residues Thr174 and Tyr176, which is required for Hog1 activation. Using commercial antibodies raised against phosphorylated human p38, we were able to detect the phosphorylation of Hog1 protein after exposure of CUR (Figure 2A).

Yeast Hog1 is a stress-activated kinase that is thought to be activated exclusively by osmotic stress. However, recent studies have implicated the role of this MAPK in mediating tolerance to a variety of stress conditions including osmotic [33], oxidative [34], heat [2], arsenic [35], and citric acid stress [3]. We have observed the phosphorylation of Hog1 in the presence of CUR (Figure 2A) and provided evidence for the activation of the HOG pathway in yeast. Hog1 activation has been most extensively characterized under conditions of hyperosmotic shock. The exposure of *S. cerevisiae* to high-osmolarity leads to a rapid (<30 min) and sustained (up to several hours depending on the severity of the conditions) phosphorylation of Hog1 protein [27-29]. To determine whether CUR induces a slow or a rapid response, phosphorylation of Hog1 was measured at intervals following the addition of 100 μM CUR. Addition of CUR induced a rapid increase in Hog1 phosphorylation, but unlike NaCl-induced Hog1 phosphorylation, it sustained for an extended period of time (Figure 2C). It has been well documented that curcumin antagonizes yeast growth by chelating iron [36]. Previously, we have demonstrated that supplementation of iron rescued the cells from the growth arrest phenotype [20]. Moreover, we also found a novel way to reset epigenetic marks back to their normal levels by iron supplementation in the presence of curcumin [20]. Hence, we were curious to understand whether CUR-induced phosphorylation of Hog1 can be restored after iron supplementation. Interestingly, the level of phosphorylated Hog1 was reduced significantly in presence of iron (Figure 3D) suggesting that the activation of MAPK Hog1 by CUR is also dependent on its iron chelating property.

Hog1 is activated through a series of phosphorylation events involving the MAPK kinase Pbs2 and several other upstream factors [33,37]. To test the role of upstream components of the HOG pathway in the cellular response to CUR, we monitored the phosphorylation of Hog1 in yeast strains carrying mutations in components of the HOG pathway. As shown in Figure 4A and B, the *pbs2*Δ mutant was defective in Hog1 phosphorylation because it is the only immediate upstream kinase for Hog1. To test the role of upstream members of the HOG pathway in the cellular response to CUR, we monitored the phosphorylation of Hog1 in yeast strains carrying mutations in the components of the HOG pathway. Interestingly, we also observed Hog1 phosphorylation defects in *SSK2, MSB2*, and *PTC2* deletion mutants, suggesting the requirement of these factors for optimal Hog1 phosphorylation in response to CUR treatment. The comprehensive CUR-induced growth inhibition analysis in the mutants incapable of phosphorylating Hog1 warrants future study, where methods outlined by the Clinical Laboratory Standards Institute (CLSI) M38-A [38] or the European Committee on Antimicrobial Susceptibility Testing (EUCAST) [39] can be used.

One of the main functions of the MAPK pathway is the regulation of transcriptional events in response to specific stimuli. Because of its phosphorylation, Hog1 gets translocated to the nucleus [30], where it targets various transcription factors, leading to upregulation of GPD1 mRNA levels [40]. Our results also revealed that CUR-induced phosphorylated Hog1 migrates to nucleus (Figure 5A). We have also demonstrated that CUR exposure causes up-regulation of *GPD1* (Figure 5B), which is required for the synthesis of a major osmolyte, glycerol. Elevated glycerol production is a prerequisite for the adaptation of *S. cerevisiae* to hyperosmotic stress [40]. Interestingly, the expression of GPD1 in response to CUR treatment requires functional Hog1 protein (Figure 5B). Notably, to the best of our knowledge, for the first time, we report that the CUR exposure leads to Hog1 phosphorylation in *S. cerevisiae* and this is followed by the activation of *GPD1* gene expression. The CUR has several molecular targets, other than Hog1. For example, CUR is known to target various cell signaling pathways such as JAK/STAT, Wnt/β-catanin, Notch, PI3K/PKB, AMPK, DNA damage checkpoint pathway [25,41-45] and many others. Here, we have identified Hog1 as an additional target of CUR. Considering Hog1 is central kinase in osmotic stress pathway, the activation of Hog1 upon CUR treatment indicates that CUR induces osmotic stress.

## Conclusions

**Figure 6:**
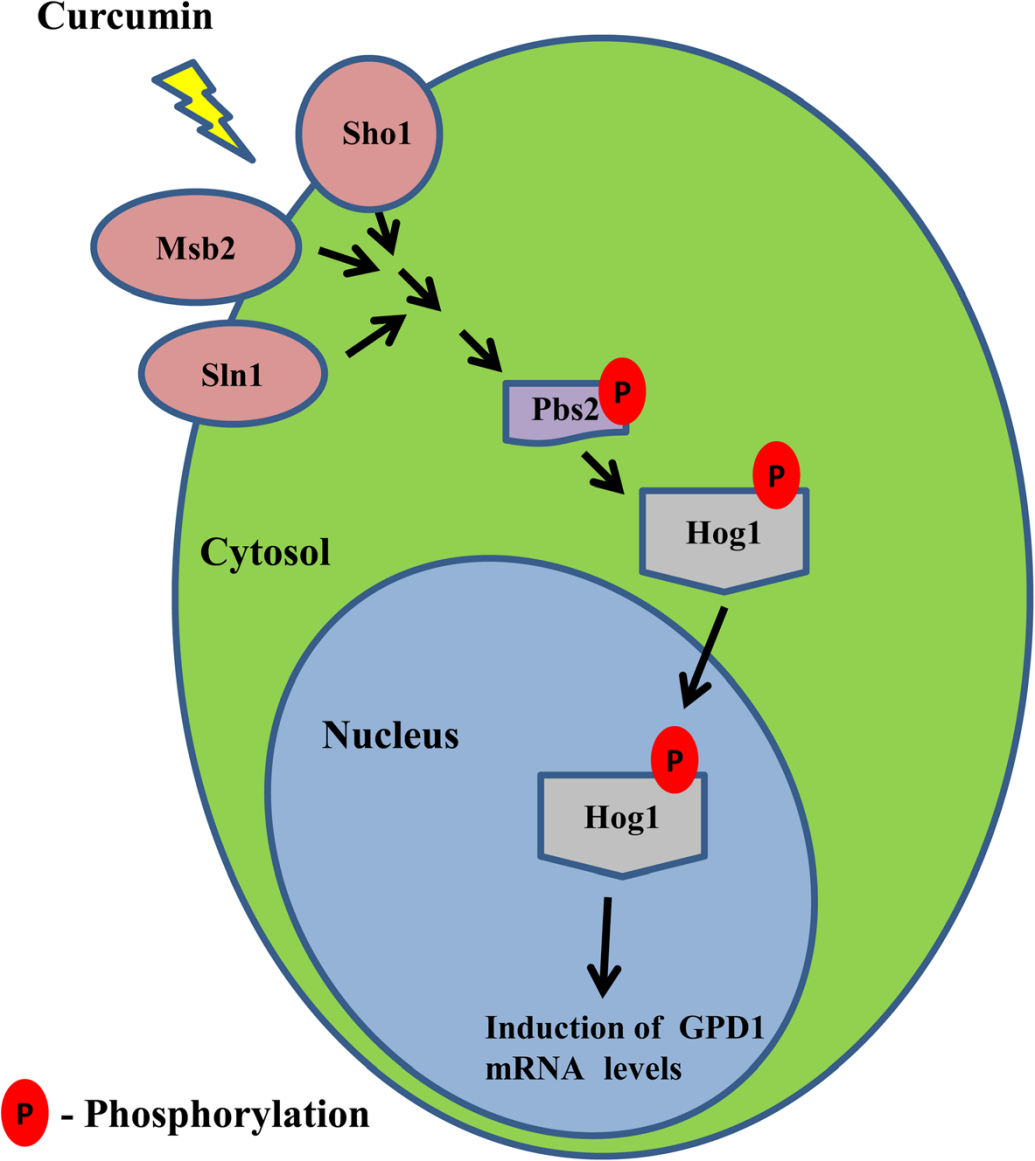
**Proposed model showing molecular mode of action of CUR on** ***S. cerevisiae.*** After entering into yeast cells CUR causes activation of osmotic stress. We have shown that CUR-induced phosphorylated Hog1 migrates to nucleus leading to up-regulation of *GPD1* mRNA levels.

Recently, the identification of bioactive dietary components has received particular interest in the field of pharmacology. Curcumin has gained immense attention for its varied therapeutic and prophylactic applications. Our results reveal new aspects of the response of *S. cerevisiae* to CUR-induced stress (Figure 6). We have identified additional functions of the Hog1 MAPK in providing transcriptional activity upon exposure to CUR. We believe that the findings presented in this work will enhance the understanding on the mode of curcumin action.
